# Molecular and Proteomic Analysis of Levofloxacin and Metronidazole Resistant *Helicobacter pylori*

**DOI:** 10.3389/fmicb.2016.02015

**Published:** 2016-12-15

**Authors:** Aimi Hanafi, Woon Ching Lee, Mun Fai Loke, Xinsheng Teh, Ain Shaari, Mojdeh Dinarvand, Philippe Lehours, Francis Mégraud, Alex Hwong Ruey Leow, Jamuna Vadivelu, Khean Lee Goh

**Affiliations:** ^1^Department of Medical Microbiology, Faculty of Medicine, University of MalayaKuala Lumpur, Malaysia; ^2^Department of Microbiology and Immunology, Yong Loo Lin School of Medicine, National University of SingaporeSingapore, Singapore; ^3^Laboratoire de Bactériologie, Université de BordeauxBordeaux, France; ^4^Institut National de la Santé et de la Recherche Médicale U853Bordeaux, France; ^5^Department of Medicine, Faculty of Medicine, University of MalayaKuala Lumpur, Malaysia

**Keywords:** *Helicobacter pylori*, levofloxacin, metronidazole, bacterial fitness, antibiotic resistance, proteome

## Abstract

Antibiotic resistance in bacteria incurs fitness cost, but compensatory mechanisms may ameliorate the cost and sustain the resistance even under antibiotics-free conditions. The aim of this study was to determine compensatory mechanisms of antibiotic resistance in *H. pylori*. Five strains of levofloxacin-sensitive *H. pylori* were induced *in vitro* to develop resistance. In addition, four pairs of metronidazole-sensitive and -resistant *H. pylori* strains were isolated from patients carrying dual *H. pylori* populations that consist of both sensitive and resistant phenotypes. Growth rate, virulence and biofilm-forming ability of the sensitive and resistant strains were compared to determine effects of compensatory response. Proteome profiles of paired sensitive and resistant strains were analyzed by liquid chromatography/mass spectrophotometry (LC/MS). Although there were no significant differences in growth rate between sensitive and resistant pairs, bacterial virulence (in terms of abilities to induce apoptosis and form biofilm) differs from pair to pair. These findings demonstrate the complex and strain-specific phenotypic changes in compensation for antibiotics resistance. Compensation for *in vitro* induced levofloxacin resistance involving mutations of *gyrA* and *gyrB* was functionally random. Furthermore, higher protein translation and non-functional protein degradation capabilities in naturally-occuring dual population metronidazole sensitive-resistant strains may be a possible alternative mechanism underlying resistance to metronidazole without mutations in *rdxA* and *frxA*. This may explain the lack of mutations in target genes in ~10% of metronidazole resistant strains.

## Introduction

*Helicobacter pylori* is a common bacterial pathogen that colonize the human stomach and is related to incidence of gastric cancer and peptic ulcer diseases (Parsonnet et al., 1991; Dhar et al., 2003). *H. pylori* infection can often be successfully eradicated with antibiotics (Heo and Jeon, 2014). However, the increasing prevalence of antibiotic resistance in *H. pylori* is a cause of concern as this is one of the most important causes of therapy failure (Graham and Fischbach, 2010). The prevalence of *H. pylori* antibiotic resistance has been associated with extensive use of antibiotics within a population (Megraud and Lehours, 2007; de Francesco et al., 2010).

The antibiotics used to treat *H. pylori* infection were mainly amoxicillin, clarithromycin, and metronidazole; these would be administered for 10–14 days in combination with an anti-secretory drug to increase the pH (Lind et al., 1999). Current recommendations for *H. pylori* treatment include the first line therapy, which is standard triple therapy consisting a combination of proton pump inhibitors (PPI), clarithromycin, and amoxicillin or metronidazole; the second line therapy will be used in the case of treatment failure, in which bismuth-based quadruple therapy or levofloxacin-containing triple therapy are recommended (Malfertheiner et al., 2007).

Levofloxacin, a fluoroquinolone, was shown to have eradicated *H. pylori* effectively (Cammarota et al., 2000). Fluoroquinolones generally target chromosome replication and in particular, DNA gyrase, which allows DNA unraveling before replication. However, the prevalence of levofloxacin resistance in *H. pylori* has been increasing worldwide (de Francesco et al., 2010); with resistance rates at 14.1% in Europe (Megraud et al., 2013), 20.6% in southeast region of China (Su et al., 2013), and 18.4% in Vietnam (Binh et al., 2013). This resistance has been associated with the point mutations occurring at positions Asn87 and Asp91 of the quinolone resistance determining region (QRDR) within *gyr*A gene (Miyachi et al., 2006; Rozen et al., 2007; Lee et al., 2011). Other mutations that have also been linked to levofloxacin resistance include mutations at positions Ala88, Ala97, and Met191 of *gyrA* and Phe438, Asp481, and Arg484 of *gyrB* (Miyachi et al., 2006; Liu et al., 2011; Teh et al., 2014).

Metronidazole, a nitroimidazole, acts as a biocidal agent by its interaction with a nitroreductase homolog, RdxA. Reduction of metronidazole results in the formation of DNA-damaging and mutagenic products (Sisson et al., 2000). Mutations in *rdxA* were shown to be the cause of *H. pylori* resistance to metronidazole (Goodwin et al., 1998). Inactivation of *rdxA* reduces the effect of nitroreductases, which comes with the decrease of conversion of metronidazole into hydroxylamine that damages bacterial DNA (Olekhnovich et al., 2009). Mutation in another gene, *frxA*, encoding for NADH flavin oxidoreductase, was also implicated in *H. pylori* metronidazole resistance (Kwon et al., 2000). FrxA, another nitroreductase of *H. pylori*, may also activate metronidazole bactericidal action although the overall effects of *frxA* mutation are still being investigated (Justino et al., 2014). Mutations, such as frameshift, missense, premature truncations, deletions, and insertions within *rdxA* and *frxA* genes, are associated with metronidazole resistance (Kwon et al., 2000; Teh et al., 2014; Binh et al., 2015). In an earlier study, it was shown that 4/37 (10.8%) of the metronidazole resistant *H. pylori* strains from Malaysia could not be attributed to mutations in *rdxA* and/or *fdxA* (Teh et al., 2014). Thus, *H. pylori* may become resistant to the antibiotic via other mechanisms.

Despite the studies on the resistance-related genes, the consequences of mutations on the physiological state of *H. pylori* are poorly understood. The impact of mutation in antibiotic resistance has been studied *in vitro* in *Streptococcus pneumoniae* for levofloxacin resistance; different resistance-encoding genes were shown to cost different levels of fitness (Rozen et al., 2007). Björkholm et al. (2001) studied the biological cost of mutation in response to clarithromycin resistance in *H. pylori*; compensatory mutation has been suggested to affect bacterial fitness. However, the effects of mutations that result in levofloxacin and metronidazole resistance have not been studied in *H. pylori*. Furthermore, antibiotic resistance-related mutations may affect bacterial virulence and survival. Implications of mutations in *H. pylori* virulence proteins involved in apoptosis (Oldani et al., 2009) and biofilm formation (Cole et al., 2004) have been studied. Maintenance of plasmids and mutated virulence genes cost energy (Martínez and Baquero, 2002). Therefore, in the absence of antibiotic selective pressure, higher level of virulence may still be maintained for the survival of bacteria could be attributed to the development of compensatory mutations (Martínez and Baquero, 2002). Analysis of *H. pylori* proteome may reveal patterns of compensatory mutations in response to metronidazole and fluoroquinolone resistance. An objective of this study was to determine the proteome of resistant *H. pylori* in response to amelioration of fitness cost as part of a compensatory response.

## Materials and methods

### Induction of levofloxacin resistant strains

*H. pylori* strains sensitive to levofloxacin were incubated at 37°C in a microaerophilic atmosphere of 10% CO_2_. They were inoculated into BHI broth with a series of levofloxacin concentrations (0.0156, 0.0313, 0.0625, 0.125, 0.25, 0.5, 1, 2, 4, 8) in a 96-well plate. After each passage, the cells would be exposed to increased concentration of levofloxacin. For example, the cells grew in the broth with 0.0156 μg/ml levofloxacin would be inoculated to the broth with 0.0313 μg/ml in the next growth passage. The bacterial suspensions were transferred to antibiotics-free chocolate agar to determine the minimum inhibitory concentration (MIC) of levofloxacin after each exposure. Stable induced-resistant strains would be grown on chocolate agar supplemented with levofloxacin to confirm resistance. The identity between resistant strains and their corresponding parental sensitive strains before induction were verified by random amplification of polymorphic DNA- polymerase chain reaction (RAPD-PCR) typing using primers 1254 and 1281 as described by Akopyanz et al. (1992) with the following modifications. The reaction mix (Thermo Fisher Scientific, USA) consisted of 1 × *Taq* buffer with KCl, 0.4 mM of each deoxynucleotide triphosphates (dNTPs), 3 mM MgCl_2_, 1 U *Taq* DNA polymerase, 0.2 μM of RAPD primer, 10 ng genomic DNA in a volume of 25 μl. The conditions for PCR amplification were denaturation at 95°C for 3 min, followed by 35 cycles of 95°C for 1 min, 38°C (for primer 1254) or 32°C (for primer 1281) for 1 min, 72°C for 2 min; and then a final extension at 72°C for 5 min.

### Screening for dual population metronidazole-sensitive and resistant strains

For *in vitro* susceptibility testing of the *H. pylori* strains, a suspension equal to the McFarland tube no. 3 was prepared for each strain in BHI broth supplemented with 2% β-cyclodextrin and 0.4% yeast extract. The cell suspension was inoculated by confluent swabbing of the surface of non-selective chocolate agar with the adjusted inoculum suspensions. Metronidazole discs were laid on the agar surface. The plates were then be incubated at 37°C under microaerophilic conditions. The breakpoint zone diameter was recorded after 48–72 h of incubation. Colonies that grew within clear zones were isolated and grown on metronidazole-supplemented chocolate agar to confirm resistance. Colonies that grew around the clear zones were isolated and grown on non-selective and metronidazole-supplemented chocolate agar to confirm susceptibility. Resistant and susceptible isolates of the same strain from the antibiotics screening were selected for dual population study. Genomic DNA of *H. pylori* was extracted for PCR amplification and sequencing of *gyrA, gyrB, rdxA*, and *frxA*, which were performed as described by Teh et al. (2014).

### *H. pylori* strains and growth

**Figure 1:**
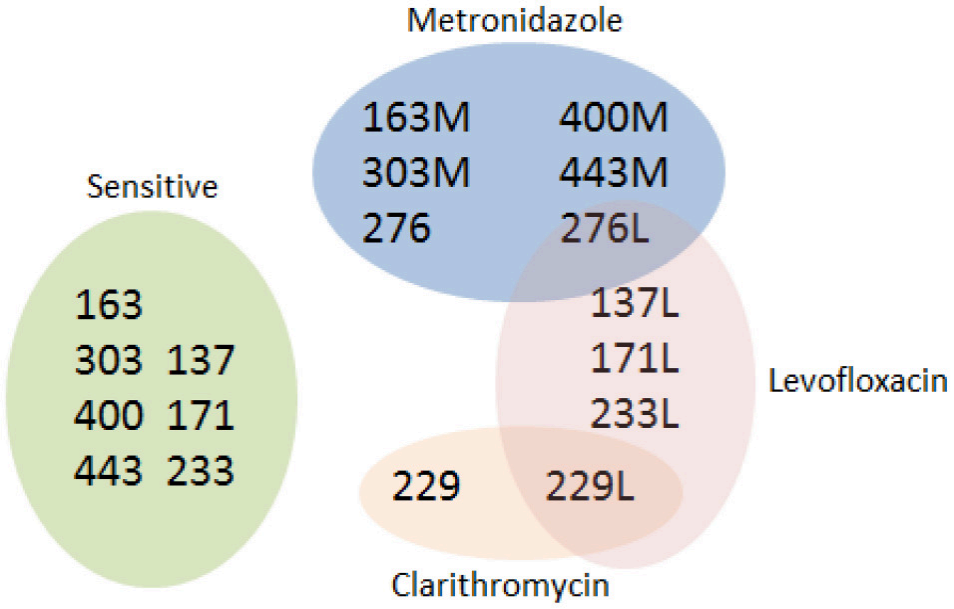
*****H. pylori*** strains according to sensitivity or resistance to metronidazole^**a**^, levofloxacin, clarithromycin^**b**^, and combined (multi-drug^**c**^) resistance**. ^a^UM276 and UM276L (levofloxacin-sensitive and resistant), UM163M, UM303M, UM400M, UM443M have naturally-occuring resistance to metronidazole. ^b^UM229 and UM229L (levofloxacin-sensitive and resistant) has naturally-occuring resistance to clarithromycin. ^c^Resistant to metronidazole, levofloxacin, and/or clarithromycin.

The *H. pylori* UM137, UM171, UM229, UM233, UM276 which consist of levofloxacin-sensitive and induced-resistant variant of each strain, and UM163, UM303, UM400, UM443 which consist of metronidazole-sensitive and naturally-occuring resistant variants of each strain were used in this study. UM276 was also resistant to metronidazole and UM229 was additionally resistant to clarithromycin. The 18 strains of *H. pylori* were categorized into sensitive and resistant groups based on their resistance to metronidazole, levofloxacin, clarithromycin, and multi-drug resistance (Figure 1). The *H. pylori* strains were cultured on chocolate agar for 3 days in microaerobic atmosphere at 37°C and the cells were harvested for protein extraction.

### Optical density and viable count

*H. pylori* strains exhibiting dual phenotypes (sensitive and resistant) were incubated respectively in BHI broth supplemented with 10% fetal bovine serum and 0.4% yeast extract in a microaerobic atmosphere at 37°C. At *t* = 0 h, the optical density (OD) of the *H. pylori* suspensions was standardized to be at the same level at 600 nm by spectrophotometry. Samples were collected every 24 h over 7 days. The OD_600_ of the cell suspension was measured by spectrophotometry and viable count was performed to compare their growth curves; a serial dilution of the incubated samples was performed and the number of colony-forming units (CFU) was assessed after each time interval.

### Apoptosis assay

AGS cells, a gastric epithelial cell line, were infected *in vitro* with *H. pylori* susceptible- and its corresponding resistant-strains at a multiplicity of infection (MOI) of 50:1 bacteria to cell (Menaker et al., 2004). *H. pylori* was removed and the infected AGS cells were harvested at time intervals of *t* = 4, 8, and 12 h. The cells were stained with Annexin V Alexa Fluor® 488 and propidium iodide according to the instructions in Tali® apoptosis kit (Thermo Fisher Scientific). Annexin V-positive and propidium iodide-negative cells were considered as apoptotic cells (Sawai and Domae, 2011). The levels of apoptosis induced by the antibiotics-resistant *H. pylori* strain and its parental susceptible strain were compared using Tali® image-based cytometer (Thermo Fisher Scientific). Apoptosis induction is an indicator of the *H. pylori* virulence activity (Cho et al., 2003).

### Biofilm measurement

Various strains of *H. pylori* cultured on chocolate agar for 3 days were harvested and incubated for another 3 days in BHI broth supplemented with 2% β-cyclodextrin and 0.4% yeast extract. The total bacterial population for the cultures was standardized at the same OD_600_ value. A volume of 2 ml of each bacterial suspension was inoculated into each well of a 24-well plate. The growth of biofilm was observed after every 24 h over 7 days of incubation at 37°C in 10% CO_2_; the bacterial suspension was aspirated and 0.1% crystal violet was added into the well. The plate was gently agitated for 30 min and the crystal violet was removed. The crystal violet-treated wells were washed with distilled water and the stained biofilm was destained with 19:1 ethanol-acetic acid. The destaining solution was collected and its absorbance was measured at OD_600_. The amount of biofilm produced by antibiotic-resistant *H. pylori* strain and its sensitive strain was compared. The ability to form biofilm indicates the ability to survive against the activity of biocidal agents (Yonezawa et al., 2010).

### Protein extraction and mass spectrometry

*H. pylori* cell pellets were lysed and its protein was extracted using Norgen's Proteospin™ total protein purification kit (Norgen Biotek Corporation, Canada). Cells were resuspended in 50 μl lysis buffer and centrifuged at 14,000 × *g* for 2 min. The supernatant was transferred into a filter column fitted in an elution tube, and centrifuged at 14,000 × *g* for 1 min. One microliter of protease inhibitor (Halt Protease and Phosphatase Inhibitor; Thermo Fisher Scientific) was added to the tube. Protein concentrations were determined by Bradford assay (Bio-Rad, USA). Each tube of protein sample was reduced with a volume of 10 mM dithiothreitol (DTT; Bio-Rad), and alkylated in the dark with a volume of 20 mM iodoacetamide (IAA; Bio-Rad). The samples were incubated at room temperature for 30 min and added with Pierce™ trypsin protease (Thermo Scientific) to digest the proteins at 1:50 trypsin:protein. The samples were then incubated at 4°C for 30 min, and subsequently at 37°C for overnight. The extracted protein was then treated for liquid chromatography mass spectrophotometry according to a previous study (Chan et al., 2015).

### Protein profiling and identification

Raw data was exported and sequencing was performed using PEAKS software (version 7.5; Bioinformatics Solution Inc., ON, Canada). The reference proteome database was *H. pylori* J99. Results were refined by using a false discovery rate (FDR) of 1%, and 1 unique peptide according to metronidazole- and levofloxacin-sensitive and resistant strain groups (Supplementary Material).

Proteins common to all strains in each group were selected (levofloxacin-sensitive, levofloxacin-resistant, metronidazole-sensitive, metronidazole-resistant) for protein expression analysis. Protein-protein interaction networks were analyzed by using STRING 10 (Szklarczyk et al., 2015). Proteins which were differentially expressed between sensitive and resistant groups were further analyzed for their functions in molecular and biological functions from UniProt database (The Uniprot Consortium, 2008) and pathways from KEGG database (Kanehisa et al., 2014), and possible roles in compensatory response.

### Independence test of resistance and genes

The up- or down-regulation of all expressed proteins in each strain was scored, and the difference between antibiotic-sensitive and -resistant strain groups was determined in terms of fold-expression. Proteins with the difference in scores of between 0.5- and 2 fold-difference were eliminated. The independence test of association between antibiotics resistance and the genes corresponding to the expressed proteins was determined by Fisher's exact test which was performed using SPSS (version 20; SPSS Inc, Chicago, USA). The antibiotics resistance in this test refers to the resistance against levofloxacin, metronidazole, clarithromycin or the combination resistance of any of the antibiotics (levofloxacin and clarithromycin, or levofloxacin, and metronidazole).

## Results and discussion

The emergence of resistant bacteria as a result of adaptability to antibiotics has no doubt been an emerging concern for human health. In turn, resistant bacteria presumably gained mutations that could ameliorate the reduced fitness as a result of its adaptation to antibiotics. These mutations were considered to be involved in compensatory mechanism that compensates for the fitness lost due to resistance (Handel et al., 2006). Compensatory mutations could occurred in regions of the genome due to selective pressure to compensate for the deleterious effects of the initial resistance mutations (Maisnier-Patin and Andersson, 2004). These mutations may be involved in any particular molecular or biological functions. A proteomic study on metronidazole-resistant *H. pylori* had examined metabolic changes in the bacteria which reported down- and up-regulation of various proteins including a protein with reductase activity (McAtee et al., 2001). The protein composition of the antibiotics resistant *H. pylori* may reveal the biological pathways involved in a compensatory response.

### Functional characteristics

**Figure 2:**
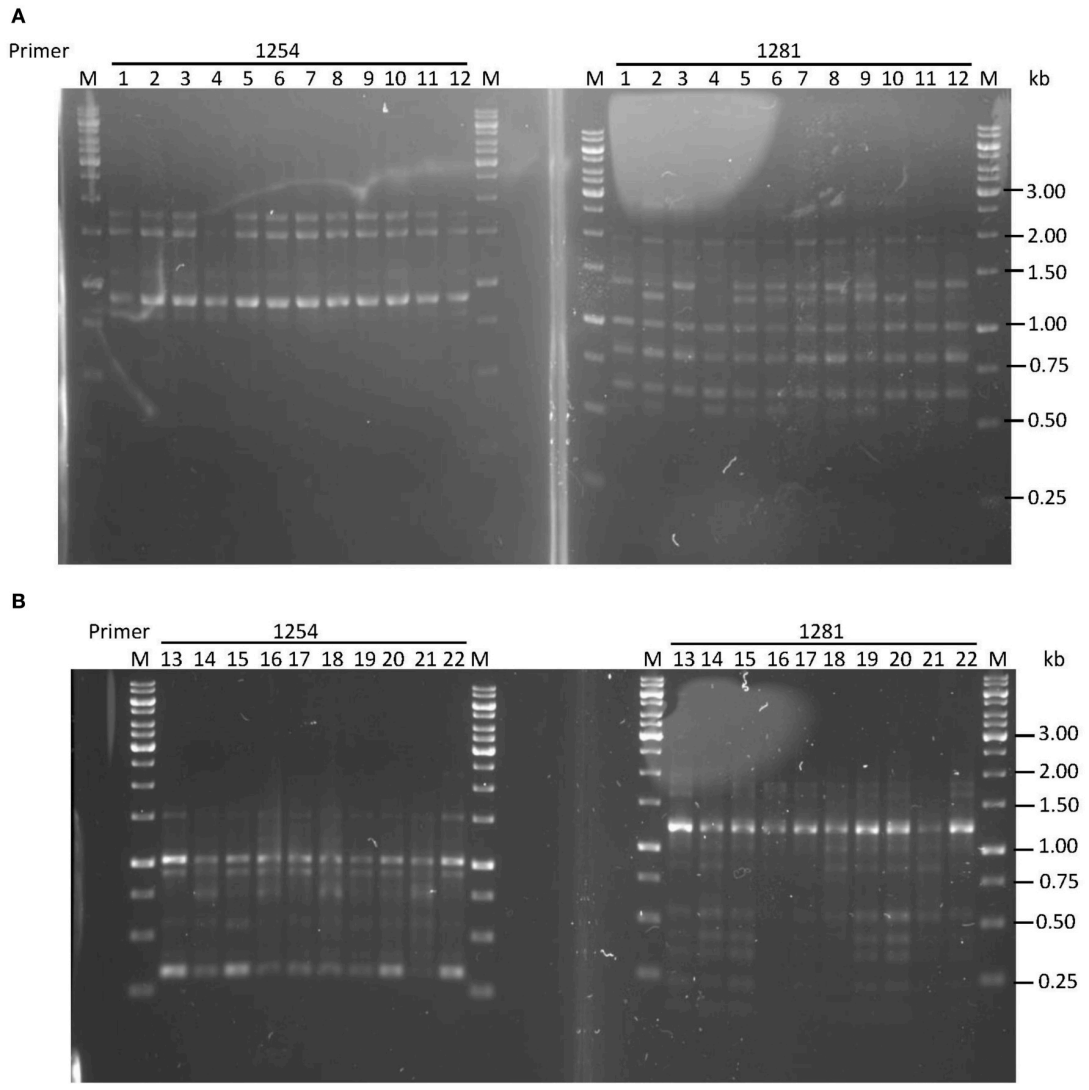
**RAPD-PCR typing of (A)** levofloxacin-induced *H. pylori* UM137 strains 1–8 sensitive, 9–12 resistant **(B)** metronidazole dual population of *H. pylori* UM163 strains 13–19 sensitive, 20–22 resistant. Primers used were 1254 and 1281, and M: 1 kb DNA ladder.

Based on MIC, strains were considered to be levofloxacin-sensitive (<1 μg/ml) or resistant (≥1 μg/ml; Chisholm and Owen, 2009); or metronidazole-sensitive (<8 μg/ml) or resistant (≥8 μg/ml; Osato et al., 2001). Five levofloxacin-sensitive *H. pylori* strains with MIC ≤ 0.125 μg/ml were successfully induced *in vitro* to become resistant with MIC > 32 μg/ml, while four pairs of naturally-occurring metronidazole-resistant *H. pylori* strains with MIC > 256 μg/ml and metronidazole-sensitive strains with MIC ≤ 0.75 μg/ml were isolated for this study (Table 1). These four pairs of metronidazole-sensitive and -resistant strains were isolated after screening through 170 *H. pylori* positive gastric tissue biopsies collected from patients seen at the Endoscopy Unit (University of Malaya Medical Centre, Kuala Lumpur, Malaysia) during the period from July 2011 to June 2014. All pairs of sensitive and resistant strains for both antibiotics were verified to be identical by RAPD-PCR genotyping (Figure 2).

**Table 1:** **MICs of ***H. pylori*** strains before and after levofloxacin-resistance ***in vitro*** induction (UM137, UM171, UM229, UM233, and UM276) and naturally-occuring metronidazole-sensitive and resistant ***H. pylori*** strains (UM163, UM303, UM400, UM443)**.

***H. pylori* Strains**	**Levofloxacin/metronidazole**	**MIC before induction/sensitive (μg/ml)**	**MIC after induction/resistant (μg/ml)**
UM137 and UM137L	Levofloxacin-resistance (induced)	0.064	>32
UM171 and UM171L	Levofloxacin-resistance (induced)	0.094	>32
UM229^b^ and UM229L	Levofloxacin-resistance (induced)	0.032	>32
UM233 and UM233L	Levofloxacin-resistance (induced)	0.125	>32
UM276^a^ and UM276L	Levofloxacin-resistance (induced)	0.125	>32
UM163 and UM163M	Metronidazole-resistance (naturally-occurring dual population)	0.5	>256
UM303 and UM303M	Metronidazole-resistance (naturally-occurring dual population)	0.75	>256
UM400 and UM400M	Metronidazole-resistance (naturally-occurring dual population)	0.5	>256
UM443 and UM443M	Metronidazole-resistance (naturally-occurring dual population)	0.75	>256

a *UM229 and UM229L (levofloxacin-sensitive and resistant) has naturally-occuring resistance to clarithromycin*.

b *UM276 and UM276L (levofloxacin-sensitive and resistant), UM163M, UM303M, UM400M, UM443M have naturally-occuring resistance to metronidazole*.

**Figure 3:**
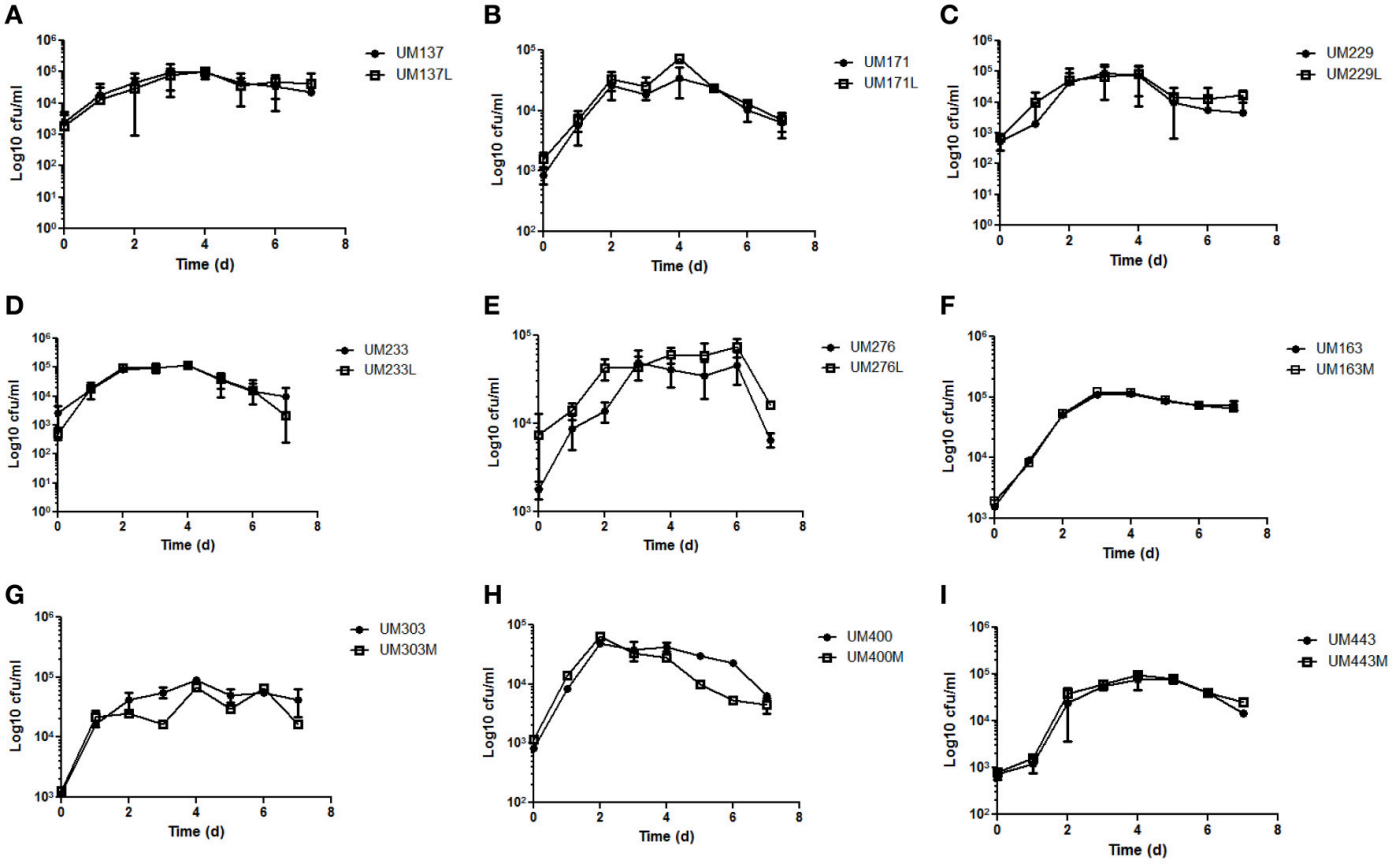
**Growth curves of (A)** UM137 **(B)** UM171 **(C)** UM229 **(D)** UM233 **(E)** UM276 **(F)** UM163 **(G)** UM303 **(H)** UM400 **(I)** UM443 *H. pylori* strains. Data represent mean ± SEM (*n* = 3).

Determination of growth rate and generation time is often used to measure fitness costs associated with antibiotic resistance (Pope et al., 2010). The growth curves of these five pairs of levofloxacin-sensitive and resistant, and four pairs of metronidazole-sensitive and resistant *H. pylori* strains were not significantly different within pairs (*p* ≥ 0.05; Figure 3). Thus, change in antibiotics resistance status did not reduce the growth fitness of the resistant strains compared to its sensitive counterparts. However, a limitation of growth curve is that the competitive fitness of sensitive strains over its resistant counterparts could not be assessed.

Among the nine pairs of sensitive and resistant strains, only three pairs of dual strains showed significant change in their ability to induce apoptosis in AGS cells within pairs (*p* < 0.05). The level of apoptotic AGS cells induced by *H. pylori* infection post-12 h showed a decrease in the metronidazole resistant strains, UM163M and UM303M, and an increase in the resistant strain, UM400M, compared to their sensitive counterparts (Table 2). In contrast, none of the levofloxacin resistant strains showed significant change in ability to induce apoptosis compared to their sensitive counterparts. Uninfected cells acted as negative control while staurosporine-treated AGS cells act as the positive control (four-fold increase in apoptosis compared to untreated AGS cells).

**Table 2:** **Fold differences of the apoptotic AGS cells population induced by ***H. pylori*** from the ratio of resistant and sensitive pair strains, post-12 h of infection**.

**Strain**	**Fold difference of apoptotic cells population (resistant:sensitive pair strain)**
UM137 and UM137L	1.33
UM171 and UM171L	1.00
UM229 and UM229L	1.33
UM233 and UM233L	0.83
UM276 and UM276L	1.25
UM163 and UM163M	0.33^*^
UM303 and UM303M	0.25^*^
UM400 and UM400M	3.50^*^
UM443 and UM443M	1.50

* *Fold difference <0.5 and >2.0 is considered to have undergone significant change*.

At population level, biofilm formation is a multicellular strategy for survival, and indirectly increases overall bacterial survival fitness. Bacterial biofilm presents as a physiological barrier at stationary growth phase against antibiotics whereby viable cells may persist due to the impenetrable structural matrix, the equilibrium of live and dead cells, or the regulation of genes expression (Normark and Normark, 2002). A majority of these strains were low-level biofilm formers, except for UM137, UM163, UM171, and UM443, and fold changes between the resistant and sensitive strains in the low-level biofilm forming pairs were not significantly different (*p* ≥ 0.05) (Table 3). Among the high-level biofilm forming strains, UM137L showed a significant decrease in biofilm formed. On the other hand, UM443M showed no significant increase of the biofilm formed over 7 days (*p* ≥ 0.05), while the biofilm formed by UM163M and UM171L were decreased compared to UM163 and UM171 respectively; the fold change did not reach statistical significance (*p* ≥ 0.05).

**Table 3:** **Average biofilm formation of ***H. pylori*** strains over 7 days of growth and the fold difference between resistant and sensitive pair strains**.

**Strain**	**Average amount of biofilm formation**	**Fold difference of the amount of biofilm formation (resistant:sensitive pair strain)**
UM137	0.744	0.240^#^
UM137L	0.178	
UM171	0.796	0.777^#^
UM171L	0.618	
UM229	0.039	3.259
UM229L	0.126	
UM233	0.158	0.184
UM233L	0.029	
UM276	0.092	1.348
UM276L	0.124	
UM163	0.420	0.723^#^
UM163M	0.304	
UM303	0.098	1.058
UM303M	0.104	
UM400	0.020	4.627
UM400M	0.091	
UM443	0.258	2.004^#^
UM443M	0.518	

# *High-level biofilm former strains*.

### Molecular characteristics

**Figure 4:**
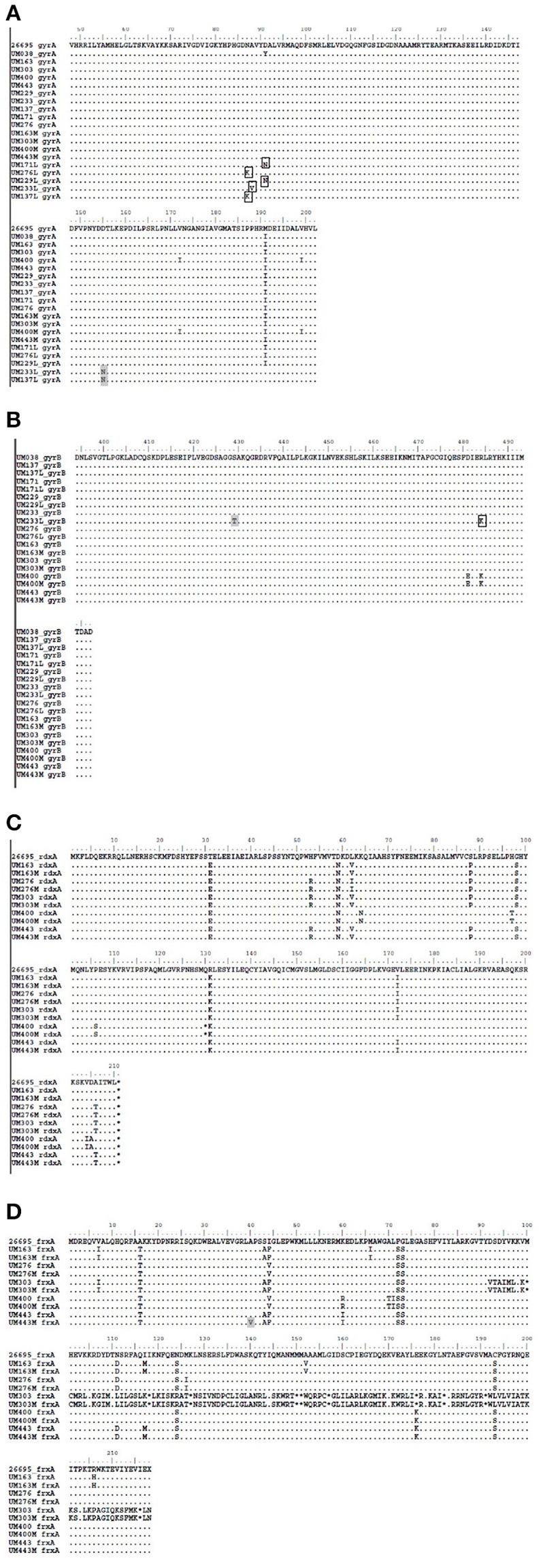
**Amino acid sequences of levofloxacin resistance-associated gene regions: (A)** *gyrA*, **(B)** *gyrB*, and metronidazole resistance-associated gene regions: **(C)** *rdxA*, **(D)** *frxA* of *H. pylori* strains. *gyrA* and *gyrB* sequences were compared to UM038 strain (positive *gyrA* mutation), and *rdxA* and *frxA* sequences were compared to a reference strain, 26695. Positions of expected mutations associated with levofloxacin are in box outlines, and positions of variations of mutation in either antibiotic-resistant strains are in gray boxes.

*GyrA* mutations occurred in the QRDR of the induced levofloxacin-resistant strains at N87K for UM137L and UM276L, A88V for UM233L, and D91N for UM171L and UM229L, D155N for UM137L and UM233L whereas substitutions S429T and R484K occurred in *gyrB* of UM233L (Figure 4). On the other hand, no specific mutations occurred in naturally-occuring metronidazole resistant strains, except for A40V in *frxA* of UM443M. Thus, *in vitro* induction of levofloxacin resistance could be explained by known *gyrA* and/or *gyrB* mutations while the naturally-occurring dual population metronidazole resistance could be caused by mutations in other genes or may involve other mechanisms.

### Inter-gene or intra-molecular protein interactions

In order to explore the underlying compensatory mechanisms adopted by antibiotic resistant *H. pylori* to maintain their overall fitness, protein profiling on the nine pairs of sensitive and resistant *H. pylori* strains was performed. All expressed proteins with significant association with levofloxacin, metronidazole, clarithromycin, and/or any combination of them (levofloxacin and clarithromycin, or levofloxacin, and metronidazole) were identified using Fisher's Exact test with *p* < 0.05 considered to be significant (Table 4).

**Table 4:** **Proteins of significant association with resistance to metronidazole, levofloxacin, and/or clarithromycin in all of the 18 ***H. pylori*** strains tested using Fisher's exact test (2-tailed, ***p*** < 0.05 is considered significant)**.

**Protein**	**Protein description/function**	**Antibiotic resistance**	**Fisher's exact test (*p*-value)**
UvrB	UvrABC system protein B	Levofloxacin	0.014
Jhp_0525	Putative protein	Levofloxacin	0.031
Jhp_0602	Putative processing protease	Levofloxacin	0.031
Jhp_0260	Putative protein	Levofloxacin	0.032
CarB	Carbamoyl-phosphate synthase large chain	Levofloxacin	0.033
ClpP	Chaperone protein	Metronidazole	0.007
Jhp_0892	Putative protein	Metronidazole	0.007
SerA (Jhp_0984)	D-3-phosphoglycerate dehydrogenase	Metronidazole	0.007
Efp (Jhp_0163)	Elongation factor P	Metronidazole	0.022
HypB (Jhp_0837)	Hydrogenase/urease nickel incorporation protein HypB	Metronidazole	0.022
IleS (Jhp_1317)	Isoleucine-tRNA ligase	Metronidazole	0.022
ProS (Jhp_0067)	Urease subunit beta	Metronidazole	0.022
RplF (Jhp_1224)	50S ribosomal protein L6	Metronidazole	0.022
RpsS (Jhp_1235)	30S ribosomal protein S19	Metronidazole	0.022
PyrF (Jhp_0005)	Orotidine 5′-phosphate decarboxylase	Metronidazole	0.031
RpsI (Jhp_0076)	30S ribosomal protein S9	Metronidazole	0.031
Jhp_0844	Flagellar basal body protein	Metronidazole, levofloxacin	0.042
Jhp_1071	Putative protein	Metronidazole, levofloxacin	0.042
Jhp_1303	Putative protein	Metronidazole, levofloxacin	0.042
CysS (Jhp_0818)	Cysteine-tRNA ligase	Metronidazole, levofloxacin	0.042

*These proteins were up-regulated in the levofloxacin and metronidazole resistant H. pylori strains*.

**Figure 5:**
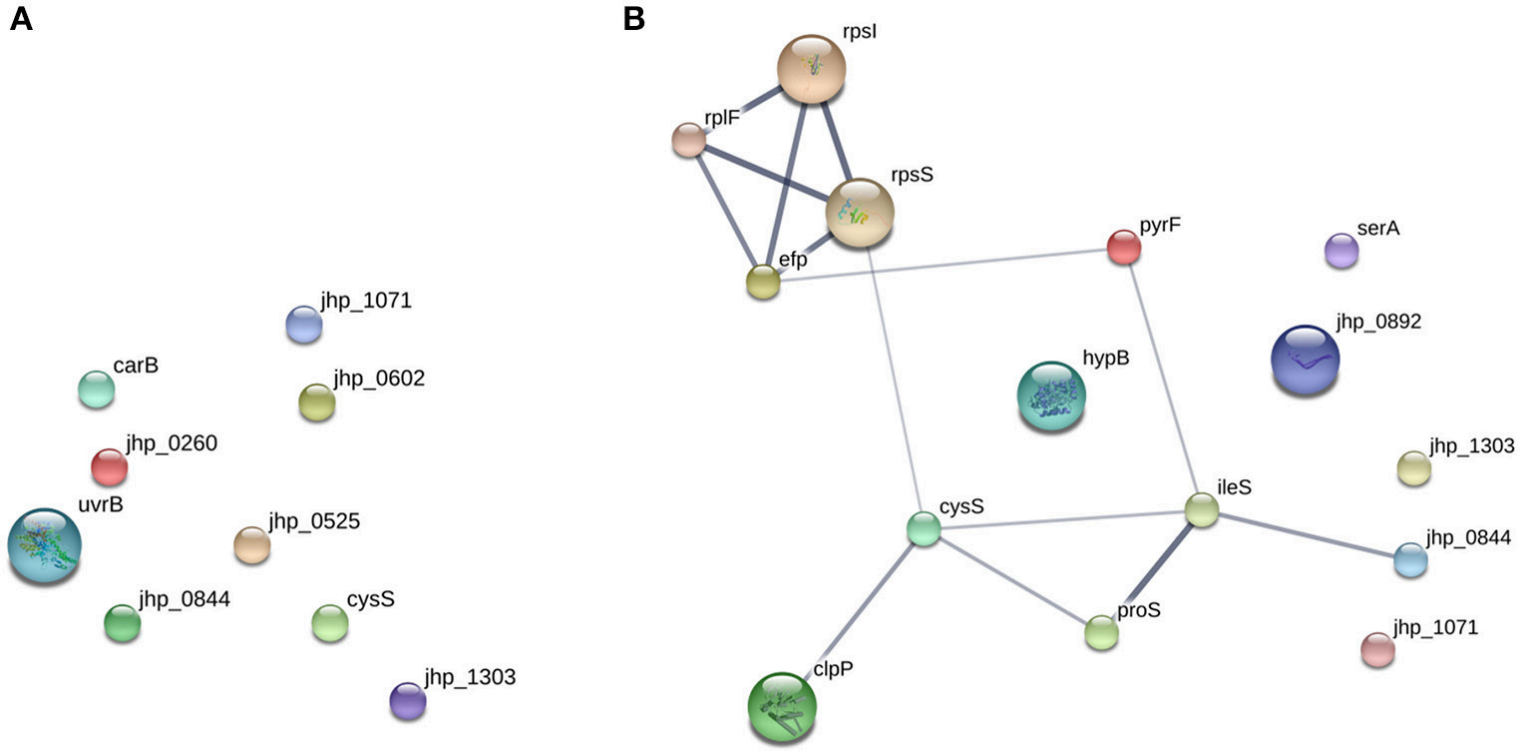
**STRING protein-protein interaction network of proteins from PEAKS refined data**. The gene names correspond with the proteins that were recovered from **(A)** levofloxacin and **(B)** metronidazole-sensitive and resistant strains.

From the prediction of possible protein-protein interactions by STRING, changes in protein expression profiles of metronidazole-resistant strains appeared to be functionally related compared to levofloxacin-resistant strains (Figure 5). Metronidazole resistance associated proteins are involved in translation, ATP binding, ligase activity, aminoacyl-tRNA editing activity, rRNA binding, or structural constituent of ribosome (Table 5). These up-regulated proteins may either represent an alternative mechanism of metronidazole resistance in a dual population environment without genetic mutations in *rdxA* and *frxA* and/ or as a compensatory mechanism underlying metronidazole resistance. The alteration in protein profile may explain the dual population phenomenon whereby some bacterial cells were more resistant to antibiotics than others despite having similar genetic makeup.

**Table 5:** **Proteins with differential expression among nine pairs of levofloxacin and metronidazole-resistant and sensitive strains**.

**Gene/Protein**	**Molecular function**	**Biological process**	**Pathway (KEGG pathway)**
*proS/*Proline–tRNA ligase	Aminoacyl-tRNA editing activity, ATP binding, proline-tRNA ligase activity	Prolyl-tRNA aminoacylation	Aminoacyl-tRNA biosynthesis (970)
*ileS/*Isoleucine–tRNA ligase	Aminoacyl-tRNA editing activity, ATP binding, isoleucine-tRNA ligase activity, zinc ion binding	Isoleucyl-tRNA aminoacylation	Aminoacyl-tRNA biosynthesis (970)
*cysS/*Cysteine–tRNA ligase	ATP binding, cysteine-tRNA ligase activity, zinc ion binding	Cysteinyl-tRNA aminoacylation	Aminoacyl-tRNA biosynthesis (970)
*rpsS/*30S ribosomal protein S19	rRNA binding, structural constituent of ribosome	Translation	Ribosome (3010)
*rplF/*50S ribosomal protein L6	rRNA binding, structural constituent of ribosome	Translation	Ribosome (3010)
*rpsI/*30S ribosomal protein S9	Structural constituent of ribosome	Translation	Ribosome (3010)
*serA/*D-3-phosphoglycerate dehydrogenase	Amino acid binding, NAD binding, phosphoglycerate dehydrogenase activity	L-serine biosynthetic process	Metabolic pathways (1100)
*pyrF/*Orotidine 5′-phosphate decarboxylase	Orodotine-5′-phosphate decarboxylase activity	“*de novo*” pyrimidine nucleobase biosynthetic process, “*de novo*” UMP biosynthetic process	Metabolic pathways (1100)
*jhp_0844/*Putative flagellar basal-body/rod/hookprotein		Bacterial-type flagellum-dependent cell motility	Flagellar assembly (2040)

*These proteins were up-regulated in the levofloxacin and metronidazole resistant H. pylori strains*.

It has been suggested that the presence of different proteins in strains with different antibiotic resistance may be attributed to epistasis (Salverda et al., 2011). The effect of compensatory response against antibiotic resistance may be due to the interactions between the primary resistance genes and the secondary mutated genes (Moore et al., 2000; Baker et al., 2013). In addition, the association of genes with the sensitive-resistant groups of different antibiotic groups may be due to the effect of either positive or negative epistasis (Schenk et al., 2013). It may also be possible that these compensatory protein responses are in the vicinity of their expressed resistance mutations in their primary or tertiary protein structure (Davis et al., 2009). This may explain the levofloxacin sensitive strains protein expression profile, which seem to be functionally random, as compared to that of the metronidazole resistant strains. Afterall, point mutations in *gyrA* and *gyrB*, which are responsible for levofloxacin resistence, also occurred at random positions along the genes. These point mutations at different positions may alter the interaction and functions of these proteins differently.

### Protein translation of antibiotic resistant strains

ProS, IleS, and CysS are aminoacyl-tRNA ligases (also known as aminoacyl-tRNA synthetases) that are involved in the synthesis of aminoacyl-tRNAs, which bind to ribosomes during the translation process (Hendrickson and Schimmel, 2003). In the current study, these aminoacyl-tRNA synthetases were found to be expressed in metronidazole resistant *H. pylori* strains (except CysS that was also expressed in levofloxacin resistant strains) but not in sensitive strains. In *Clostridium difficile*, aminoacyl-tRNA synthetases, CysS, and SerS, were highly expressed in bacterial strains that exhibit resistance to metronidazole (Moura et al., 2014). Interestingly, it was also shown that these aminoacyl-tRNA synthetases were not detected the same metronidazole-resistant *C. difficle* when cultured in the presence of the antibiotic (Moura et al., 2014). In the current study, in order to compare the protein profiles between sensitive and resistant pair of strains, bacteria for proteomics profiling were cultured in the absence of antibiotics.

Ribosomal proteins (RpsS, RplF, and RpsI) were expressed in metronidazole resistant *H. pylori* strains but not in sensitive strains.

Different expressions of ribosomal proteins have been associated with bacterial fitness (Lind et al., 2010). It has been shown that the alteration of *rplF* gene (50S ribosomal protein L6) in *Escherichia coli* mutants interrupted cell proliferation since this protein plays an important role in *E. coli* 50S subunit assembly (Shigeno et al., 2016). Similarly, down-regulation of RpsI (30S ribosomal protein S9) has been associated with decreasing rate of protein synthesis in *E. coli* (Dabbs, 1983; Hoang et al., 2004).

Elongation factor P (EFP), which is a prokaryotic protein translation factor required for efficient peptide bond synthesis on 70S ribosomes (Blaha et al., 2009), was also associated with metronidazole resistance in *H. pylori* further suggesting that protein translation were up-regulated in antibiotic resistant strains. ATP-dependent caseinolytic proteases that acts both as a chaperone and as an ATPase driving the degradation of damaged or mis-made proteins has been demonstrated to play a role in modulation susceptibility to antibiotics causing protein damage and/or oxidative stress (Loughlin et al., 2009). From our results, ClpP was found to be expressed in metronidazole resistant strains indicating that the ability to degrade non-functional proteins is an important part of the mechanism. Thus, we hypothesized that in a dual population environment, bacterial cells with more efficient translation machinery were better at overcoming the action of metronidazole, which targets bacterial DNA. This may represent an alternative mechanism that could be used by *H. pylori* to counter the actions of metronidazole. Unlike mutations in *frxA* and *rdxA*, this alternative mechanism of metronidazole resistance will be harder to detect.

### Metabolism of antibiotic resistant strains

SerA (D-3-phosphoglycerate dehydrogenase) is an enzyme that is involved in the conversion of 3-phosphoglycerate into 3-phosphohydroxypyruvate, one of the steps in serine biosynthesis pathway (Shimizu et al., 2008). PyrF (orotidine 5′-phosphate decarboxylase) is known to be an efficient enzyme in the catalyzation of orotidine 5′-monophosphate to uridine 5′monophosphate (Harris et al., 2000) and plays a role in pyrimidine metabolism (Capone et al., 2007). Bacteria has been suggested of undergoing different levels of metabolism when proteins involved in amino acid metabolism are up-regulated (Sauer et al., 2002; Drenkard, 2003). In this study, SerA and PyrF were shown to be associated with metronidazole resistance in *H. pylori*. Alteration in metabolic pathways of antibiotic resistant strains may be a compensatory response in order to balance the energy production associated with antibiotic resistance. The higher cost of fitness needs to be compensated thus enabling a higher survival rate of the bacteria.

## Conclusion

Growth rates of both induced and naturally occurring levofloxacin and metronidazole resistant strains of *H. pylori* strains were comparable with their respective parental strains indicating that the acquisition of antibiotic resistance was well-compensated with no loss of growth fitness. However, virulence and biofilm forming abilities were altered in certain antibiotic resistant strains compared to their parental strains suggesting that gain or loss of virulence and survival fitness in *H. pylori* is strain specific reflecting the genetic diversity among *H. pylori* strains. Our results may also suggest that in a dual population environment, higher protein translation, and non-functional protein degradation capabilities may be used by *H. pylori* to as an alternative strategy to counter the action of metronidazole in the absence of mutations in *frxA* and *rdxA*. In order to maintain energy balance and fitness, metabolic pathways may be altered in compensation. In comparison to metronidazole, compensation for levofloxacin resistance, which involves mutations in *gyrA* and *gyrB*, the compensatory mechanism was functionally more random as suggested by their proteomic profiles.

## Author contributions

PL, FM, ML, and KG conceived the work. AH, AS, and MD conducted the levofloxacin resistance induction and AH performed the growth, apoptosis, biofilm formation assays. XT performed the PCR amplification of the resistance-associated genes. ML and AH performed the protein profiling and bioinformatics analysis. AH, WL, ML, AL, and JV contributed to the preparation of the manuscript. All authors participated in the critical review of this manuscript.

## Funding

This project was funded by the Ministry of Education, Malaysia through University of Malaya High Impact Research Grant UM.C/625/1/HIR/MoE/CHAN/13/2 (Account No. H-50001-A000032) and University of Malaya Research Grant (UMRG) Reference No. RP016B-13HTM.

### Conflict of interest statement

The authors declare that the research was conducted in the absence of any commercial or financial relationships that could be construed as a potential conflict of interest.
